# Lost in Translation? Bridging the Gap in Communication Through Experiential Learning in Psychiatry

**DOI:** 10.1192/bjo.2025.10081

**Published:** 2025-06-20

**Authors:** Madeeha Bandukda, Hussain Bux, Natasha Donovan

**Affiliations:** North East London NHS Foundation Trust, London, United Kingdom

## Abstract

**Aims:** Much like a doctor’s diagnostic or procedural skills, communication skills are crucial in shaping patient outcomes. Poor communication in psychiatry through use of medical jargon, failure to validate concerns or a lack of empathy, can have far-reaching consequences including breakdown of the doctor-patient relationship, disengagement from treatment potentially resulting in a mental health crisis and future distrust of medical professionals. Concerningly, research suggests that without targeted training, medical students’ communication skills and empathy declines during their degree. However, despite its importance, training in communication skills in psychiatry is often underrepresented in the medical curriculum. This workshop aimed to bridge this gap, equipping students with communication skills for mental health settings, improving student confidence and ultimately enhancing patient-centred care.

**Methods:** This workshop was co-developed by doctors in psychiatry at different stages of training, education fellows and patient actors. The aim was to integrate both clinical and communication expertise. Workshops were primarily delivered face to face, with one session trialled online. There were between 24–28 students in attendance for each workshop. The students were fourth year medical students from Queen Mary University, London, on placement in psychiatry at North East London NHS Foundation Trust. Forum theatre simulation techniques were used by facilitators to role play a doctor-patient consultation and encourage students to interact and actively reflect. Students then worked in groups and practiced explaining common psychiatric diagnoses and management plans to a simulated patient or relative.

**Results:** Pre- and post-session questionnaires were completed by students. Prior to the workshop, 80–92% of students reported lacking confidence in explaining a psychiatric diagnosis to a patient and 73–94% felt unprepared to discuss a psychiatric management plan. Whereas following the workshop, 72–80% felt quite or very confident explaining a psychiatric diagnosis and 82–95% reported reduced anxiety around communicating with patients in mental health settings; 91–100% rated the session as useful, engaging, and thought-provoking.

**Conclusion:** This workshop notably improved medical students’ subjective confidence in communication in mental health settings. By integrating experiential learning, real-time feedback, and role-plays, students developed essential skills for communication in psychiatry. The overwhelmingly positive feedback from students supports the need for structured communication training in medical curricula. Future sessions should include follow-up assessments to evaluate long-term skill retention and could expand to include other important areas of communication such as multidisciplinary team communication and conflict management.

